# Blastomere nucleation: Predictive factors and influence of blastomere with no apparent nuclei on blastocyst development and implantation

**DOI:** 10.5935/1518-0557.20180028

**Published:** 2018

**Authors:** Amanda Souza Setti, Rita Cássia Sávio Figueira, Daniela Paes de Almeida Ferreira Braga, Assumpto Iaconelli Jr., Edson Borges Jr.

**Affiliations:** 1 Fertility Medical Group, São Paulo - SP, Brazil; 2 Instituto Sapientiae - Centro de Estudos e Pesquisa em Reprodução Humana Assistida, São Paulo - SP, Brazil

**Keywords:** Blastocyst, embryo, ICSI, implantation, nuclei

## Abstract

**Objective:**

To investigate whether embryos presenting blastomere(s) with no apparent nucleus (BNAN) on days 2 and 3 are more likely to fail to develop into blastocysts, hatch and implant.

**Methods:**

A total of 5705 zygotes obtained from 743 intracytoplasmic sperm injection (ICSI) cycles were analyzed. The presence and incidence of BNAN on days 2 and 3 of embryo development were recorded and then associated with ICSI outcomes.

**Results:**

The occurrence of BNAN on day 2 of embryo development was determinant to the decreased odds of blastocyst formation (OR: 0.57, CI: 0.50-0.65), quality (OR: 0.56, CI: 0.43-0.73) and hatching status (OR: 0.66, CI: 0.50-0.87). The presence of BNAN on day 3 of embryo development was determinant to the decreased odds of blastocyst formation (OR: 0.67, CI: 0.58-0.78) and hatching status (OR: 0.61, CI: 0.45-0.83). The occurrence of BNAN on day 2 of embryo development was determinant to the decreased odds of blastocyst implantation (OR: 0.50, CI: 0.27-0.94).

**Conclusion:**

The presence of BNAN on day 2 or day 3 reduces development to blastocyst stage, hatching and implantation. Careful nuclear observation, taking into account the absence of blastomere nucleus, should be part of the strategies used for embryo selection.

## INTRODUCTION

After fertilization, the human zygote divides by mitoses into several smaller cells called blastomeres. This process of division, known as cleavage, is a period of intense DNA replication and cell division, with no discernible increase in overall cellular size. Cytoplasmic division occurs after nuclear replication. Finally, each blastomere nucleus should be subjected to a different cytoplasmic environment (Veeck, 1999; Elder & Dale, 2000).

In many embryo-grading systems, blastomere nucleation is an important criterion in embryo selection (Ambroggio *et al*., 2011). The nucleation status is defined as the presence or absence of nuclei in the blastomeres in the cleavage-stage embryo. Ideally, the nucleation status of each blastomere should be recorded as mononucleated, multinucleated or no visible nucleus.

The nucleus is considered normal, or mononucleated, when a single nucleus is visible (Prados *et al*., 2012). This nucleation status has been pointed as a strong predictor of pregnancy in IVF (Palmstierna *et al.,* 1998). Multinucleation, which is defined as the presence of more than one nucleus in at least one blastomere of the embryo (Van Royen *et al*., 2003), is the most studied nucleation status and it is related to increased genetic disorders of the embryo, lower blastocyst formation, lower implantation potential, increased risk of spontaneous abortion and lower live birth rates (Kligman *et al*., 1996; Van Royen *et al*., 2003; Scott *et al*., 2007; Ergin *et al*., 2014).

Usually, when multinucleation is not found, the blastomere is considered normal, thus disregarding the significance, if any, of an absent nucleus. Palmstierna *et al*. (1998) reported that visible mononucleated blastomeres exhibited high predictive value for pregnancy in IVF cycles. Similarly, in a subsequent study, the visualization of four mononucleated blastomeres in a four-cell embryo predicted a statistically significant higher implantation rate than in cases where not all four blastomeres were mononucleated (Saldeen & Sundström, 2005). Additionally, Moriwaki *et al*., (2004) reported lower implantation rates when embryos presenting blastomere(s) with no apparent nucleus (BNAN) were transferred, as compared to those obtained with the transfer of embryos presenting only mononucleated blastomeres.

The objective of this study was to investigate if embryos presenting BNAN on the second or third day of development are more likely to fail to develop into blastocysts, hatch, and implant.

## MATERIALS AND METHODS

### Experimental design, patients, inclusion and exclusion criteria

This cross-sectional study included data from patients undergoing ICSI from July 2011 to June 2014 in a private university-affiliated IVF center located in Brazil. The inclusion criteria were as follows: Patients undergoing ICSI with fresh embryo transfer performed on day 5 of development.

All patients signed a written informed consent form and the local Institutional Review Board approved the study.

### Controlled ovarian stimulation

Ovarian stimulation was achieved by the administration of recombinant follicle-stimulating hormone (Gonal-F^®^, Serono, Geneve, Switzerland) and gonadotropin-releasing hormone (GnRH) antagonist, cetrorelix acetate (Cetrotide; Serono Laboratories, Geneva, Switzerland). Ovulation was triggered by the injection of recombinant human chorionic gonadotrophin (Ovidrel™, Serono, Geneve, Switzerland).

### Laboratory procedures

ICSI was performed according to Palermo *et al*. (1992). Fertilization was confirmed by the presence of two pronuclei (PN) and the extrusion of the second polar body approximately 17 hours after ICSI.

Embryos were morphologically evaluated on days 1 (17h±1h post ICSI), 2 (44h±1h post ICSI), 3 (68h±1h post ICSI) and 5 (116h±2h post ICSI) of development. The presence of BNAN on days 2 and 3 of embryo development was recorded.

To evaluate blastocyst morphology, the embryos were given a numerical score from one to six, based on their degree of expansion and hatching status, as follows: 1, an early blastocyst with a blastocele that is less than half the volume of the embryo; 2, a blastocyst with a blastocele that is greater than half the volume of the embryo; 3, a full blastocyst with a blastocele that completely filled the embryo; 4, an expanded blastocyst; 5, a hatching blastocyst; and 6, a hatched blastocyst. The ICM of full, expanded, hatching, and hatched blastocysts was classified as either high quality (tightly packed with many cells) or low quality (loosely grouped with several or few cells). Similarly, the TE was also classified as either high quality (many cells forming a cohesive epithelium) or low quality (few cells forming a loose epithelium or very few cells) (Alpha Scientists in Reproductive Medicine and ESHRE Special Interest Group of Embryology, 2011).

Embryo transfer was performed on day 5 of development using a soft catheter with transabdominal ultrasound guidance. One to four embryos were transferred per patient, depending on embryo quality and maternal age.

### Data analysis and statistics

Regression analyses were used to investigate the influence of: (i) maternal and paternal ages, total dose of FSH administered, estradiol levels on the day of hCG administration and retrieved oocytes on the occurrence (the presence of at least one BNAN in the embryo) and the incidence of BNAN per embryo (number of BNAN divided by the total number of cells in the embryo) on days 2 and 3 of embryo development.

Binary regression analysis was used to investigate the influence of the occurrence and incidence of BNAN on days 2 and 3 of embryo development, on the formation, quality and hatching status of blastocysts on day 5 of development, and implantation chance. For the association of BNAN and implantation, only cycles in which none (0%) or all the embryos transferred had implanted (100%) were included in the analysis. Results are expressed as odds ratio (OR) with 95% confidence intervals (CI) or regression coefficients (r) and *p*-values.

A *p*<0.05 was considered statistically significant. Data analyses were carried out using the Minitab^®^ version 17 statistical package.

## RESULTS

A total of 743 ICSI cycles performed in 583 patients were included in the analysis. Single embryo transfer was performed in 51 cases (6.9%), double in 484 cases (65.1%), triple in 196 cases (26.4%), and quadruple in 12 cases (1.6%). Full descriptive analysis of the included cycles is depicted in Table 1.

**Table 1:** Descriptive analysis of patients' demographics and ICSI cycles' outcomes.

Variables	N	Mean	SD	Range
*Patients' demographics*				
Maternal age (y-old)	583	34.3	4.1	21-45
Paternal age (y-old)	583	37.2	5.6	25-63
Total FSH administered (IU)	-	2279	605	600-4950
Estradiol level (pg/mL)	-	2124	1538	125-10000
*COS outcomes*				
Aspirated follicles	14905	20.1	10.6	2-64
Retrieved oocytes	10868	14.6	8.1	1-5
Mature oocyte rate (%)	8072/10868	74.3	-	-
Injected oocytes	7609	10.2	5.0	1-36
*Laboratorial outcomes*				
Fertilization rate (%)	5705/7609	75.0	-	-
Blastocyst formation rate (%)	3147/5705	55.2	-	-
Transferred embryos	1639	2.2	0.6	1-4
*Clinical outcomes*	**N**	**%**		
Clinical pregnancy rate (%)	308/743	41.5		
Miscarriage rate (%)	48/308	15.6		
Implantation rate (%)	407/1639	24.8		

Note: SD, standard deviation; IU, international units; COS, controlled ovarian stimulation.

### Incidence of BNAN

A total of 5705 zygotes were formed. On day 2 of development, 1295 embryos presented with BNAN (22.7%). The mean number of BNAN per embryo was 1.7±0.9 (range: 1-7). The mean incidence of BNAN per embryo was 50.1%±28.2% (range: 14.3-100). A total of 913 embryos (70.5%) had more than 25% of BNAN.

On day 3 of development, 958 embryos presented with BNAN (16.8%). The mean number of BNAN per embryo was 1.8±0.9 (range: 1-7). The mean incidence of BNAN per embryo was 25.9%±15.3% (range: 8.3-100). A total of 329 embryos (34.3%) had more than 25% of BNAN.

### Predictive factors of BNAN

Maternal and paternal ages, the total dose of FSH administered the estradiol levels on the day of hCG administration and the number of retrieved oocytes were not associated with the occurrence of BNAN or the incidence of BNAN on days 2 and 3 of development (Table 2). The occurrence of BNAN on day 2 of embryo development was determinant of the occurrence of BNAN on day 3 of embryo development (OR: 2.51, CI: 2.16-2.91).

**Table 2:** Binary and linear regression analysis' results for the predictive factors of BNAN on days 2 and 3 of embryo development.

**Predictive variables**	**Response variables**			
	**Occurrence of BNAN on D2**		**Incidence of BNAN on D2**	
	**OR**	**CI**	**r**	***p*-value**
Maternal age (y-old)	0.99	0.98-1.01	-0.2329	0.250
Paternal age (y-old)	1.01	0.99-1.02	0.2807	0.055
Total FSH administered (IU)	1.00	1.00-1.00	-0.0016	0.222
Estradiol level (pg/mL)**	1.00	1.00-1.00	0.0008	0.113
Retrieved oocytes**	1.01	1.00-1.02	-0.0117	0.895
	**Occurrence of BNAN on D3**		**Incidence of BNAN on D3**	
	**OR**	**CI**	**r**	***p*-value**
Maternal age (y-old)	0.99	0.98-1.01	0,0177	0.884
Paternal age (y-old)	1.00	0.99-1.01	-0,03926	0.666
Total FSH administered (IU)	1.00	1.00-1.00	0,0013643	0.096
Estradiol level (pg/mL)**	1.00	1.00-1.00	-0,0000579	0.854
Retrieved oocytes**	1.02	1.00-1.03	0,06099	0.240

Note: BNAN, blastomere with no apparent nucleus; D2, day 2 of embryo development; D3, day 3 of embryo development; OR, odds ratio; CI, confidence intervals; r, regression coefficient; IU, international units.

### Influence of BNAN on blastocyst development

On day 5 of embryo development, 3147 blastocysts were obtained from 5705 zygotes (55.2%), and 1808 blastocysts were of high quality (57.4%). A total of 477 blastocysts were hatching or fully hatched (15.2%).

The occurrence of BNAN on day 2 of embryo development was determinant to the decreased odds of blastocyst formation (OR: 0.57, CI: 0.50-0.65), quality (OR: 0.56, CI: 0.43-0.73) and hatching status (OR: 0.66, CI: 0.50-0.87). The occurrence of BNAN on day 3 of embryo development was determinant to the decreased odds of blastocyst formation (OR: 0.67, CI: 0.58-0.78) and hatching status (OR: 0.61, CI: 0.45-0.83) (Table 3).

**Table 3:** Binary Regression analysis' results for the association between BNAN on days 2 and 3 of embryo development and blastocyst formation, quality and hatching status.

Predictive variables	Response variable	
	Blastocyst formation	
	OR	CI
Occurrence of BNAN on D2	0.57	0.50-0.65*
Incidence of BNAN on D2	1.00	0.99-1.00
Embryo with >25 % BNAN on D2	0.61	0.47-0.78*
Occurrence of BNAN on D3	0.67	0.58-0.78*
Incidence of BNAN on D3	0.97	0.95-1.00
Embryo with >25 % BNAN on D3	0.53	0.39-0.72*
	**Blastocyst quality**	
	**OR**	**CI**
Occurrence of BNAN on D2	0.56	0.43-0.73*
Incidence of BNAN on D2	1.01	1.00-1.02
Embryo with >25 % BNAN on D2	1.27	0.79-2.06
Occurrence of BNAN on D3	0.85	0.62-1.16
Incidence of BNAN on D3	1.00	0.98-1.02
Embryo with >25 % BNAN on D3	0.69	0.37-1.29
	**Blastocyst hatching status**	
	**OR**	**CI**
Occurrence of BNAN on D2	0.66	0.50-0.87*
Incidence of BNAN on D2	0.99	0.98-1.00
Embryo with >25 % BNAN on D2	0.44	0.26-0.73*
Occurrence of BNAN on D3	0.61	0.45-0.83*
Incidence of BNAN on D3	0.97	0.94-1.00
Embryo with >25 % BNAN on D3	0.76	0.38-1.54

Note: BNAN, blastomere with no apparent nucleus; D2, day 2 of embryo development; D3, day 3 of embryo development; OR, odds ratio; CI, confidence intervals;

* statistically significant.

When comparing embryos with ≤25% and >25% of BNAN, embryos with >25% BNAN on days 2 were less likely to develop into blastocysts (OR: 0.61, CI: 0.47-0.78) and to hatch (OR: 0.44, CI: 0.26-0.73), and embryos with >25% BNAN on day 3 were less likely to develop into blastocysts (OR: 0.53, CI: 0.39-0.72).

### Influence of BNAN on implantation

Out of 1639 embryos transferred in all included cycles, 767 blastocysts were transferred to patients who had 0% (351 ICSI cycles) or 100% (83 ICSI cycles) implantation rates. A total of 618 blastocysts were transferred in the 0% implantation rate group (0% IR group) and 149 blastocysts were transferred in the 100% implantation group (100% IR group). Comparison of patients' demographics and ICSI cycles' outcomes between 0% IR and 100% IR groups are shown in Table 4. There were significant differences between the 0% IR and 100% IR groups regarding the maternal (34.4±3.9 years and 32.3±4.1 years, *p*<0.001) and paternal ages (37.3±5.4 years and 35.3±5.4 years, *p*<0.001), and the total dose of FSH administered (2,279±642 IU and 2,179±614 IU, *p*=0.031).

**Table 4:** Descriptive analysis of patients' demographics and ICSI cycles' outcomes in patients with 0% or 100% implantation rate

Variables	0% IR group (n=618)	100% IR group (n=149)	*p*-value
*Patients' demographics*			
Maternal age (y-old)	34.4±3.9	32.3±4.1	<0.001
Paternal age (y-old)	37.3±5.4	35.3±5.4	<0.001
Total FSH administered (IU)	2279±642	2179±614	0.031
Estradiol level (pg/mL)	2319±1593	2717±1942	0.108
*COS outcomes*			
Aspirated follicles	22.1±11.3	21.6±9.8	0.809
Retrieved oocytes	15.8±8.1	15.9±7.5	0.591
Mature oocyte rate	77.1±15.7	76.3±13.8	0.474
Injected oocytes	11.2±5.2	10.9±4.8	0.730
*Laboratorial outcomes*			
Fertilization rate	79.6±15.6	79.9±17.8	0.318

Note: IR, implantation rate; IU, international units.

Out of the 767 blastocysts transferred, 663 derived from embryos with mononucleated blastomeres on day 2 of development and 104 from embryos with BNAN. More specifically, in the 0% IR group, there were 526/618 mononucleated embryos on day 2 (85.1%), 92/618 embryos with BNAN on day 2 (14.9%), 536/618 mononucleated embryos on day 3 (86.7%), and 82/618 embryos with BNAN on day 3 (13.3%). In the 100% IR group, there were 137/149 mononucleated embryos on day 2 (91.9%), 12/149 embryos with BNAN on day 2 (8.1%), 137/149 mononucleated embryos on day 3 (91.9%), 12/149 embryos with BNAN on day 3 (8.1%).

The implantation rate was significantly different when blastocysts derived from embryos with mononucleated and BNAN were transferred (20.7% and 11.5%, *p*=0.029). On day 3 of embryo development, 673 embryos showed mononucleated blastomeres and 94 had BNAN. Despite not being significantly different, the implantation rate tended to decrease when blastocyst derived from embryos with BNAN on day 3 were transferred (20.4% and 12.8%, *p*=0.081).

Twenty-six of 767 blastocysts transferred derived from embryos with BNAN on both days 2 and 3 (3.4%). The implantation rate of these blastocysts was significantly lower in comparison to mononucleated embryos (3.8% and 20.7%, *p*=0.032).

Binary regression analysis for the association between BNAN on days 2 and 3 of development and the implantation chance in patients with 0% or 100% implantation rate were adjusted for maternal and paternal ages and for the total dose of FSH administered as these variables were significantly different between the groups. The occurrence of BNAN on day 2 of embryo development was determinant to the decreased odds of blastocyst implantation (OR: 0.50, CI: 0.27-0.94). There were no significant associations between the likelihood of blastocyst implantation and the incidence of BNAN on D2 (OR: 1.00, CI: 0.99-1.02), embryos with >25% BNAN on D2 (OR: 1.43, CI: 0.50-4.11), occurrence of BNAN on D3 (OR: 0.87, CI: 0.56-1.37), incidence of BNAN on D3 (OR: 0.98, CI: 0.95-1.02), and embryos with >25 % BNAN on D3 (OR: 0.56, CI: 0.20-1.59).

## DISCUSSION

In the last decades, embryo selection for transfer has been based on critical assessment of morphological parameters during embryo development *in vitro*. In the present study, we hypothesized that a different approach of embryo nuclearity evaluation, taking into consideration the occurrence of BNAN, is associated with embryo developmental competence and implantation. Our results showed that the presence of at least one BNAN on day 2 of embryo development reduces blastocyst formation in 43%, blastocyst quality in 44% and blastocyst hatching in 34%; and the presence of at least one BNAN on day 3 reduces blastocyst formation in 33% and blastocyst hatching in 39%. Moreover, taking into consideration only cycles in which none (0%) or all (100%) the blastocysts transferred had implanted, the implantation rate was significantly higher when blastocysts derived from mononucleated embryos on day two were transferred as compared to blastocysts derived from embryos with BNAN. The implantation rate tended to decrease when blastocysts derived from embryos with BNAN on day 3 were transferred as compared to blastocysts derived from mononucleate embryos. Additionally, regression analysis showed that the presence of at least one BNAN on day 2 of embryo development reduced implantation chance in 50%. Our results not only bring novel information regarding the association between BNAN and blastocyst development, hatching and implantation potential, but also corroborate previous findings (Palmstierna *et al*.,1998; Moriwaki et al., 2004; Saldeen & Sundström, 2005).

In this study, we found that the incidence of BNAN per embryo was almost 2-fold higher in day-2 embryos as compared to day-3 embryos. This finding corroborates previous information that nuclear observations are more rewarding on day-2 embryos as compared to day-3 embryos (Van Royen *et al*., 2003; Prados *et al*., 2012). It has been suggested that embryos on day 2 of development have fewer and larger blastomeres as compared to embryos on day 3 of development, which favors optical accessibility (Van Royen *et al*., 2003; Prados *et al*., 2012). In fact, 30% of embryos with multinucleation in the 2-cell stage did not show multinucleation in the 3 to 8-cell stage (Staessen & Van Steirteghem, 1998).

We also found that the occurrence of BNAN on day-2 embryos is predictive of the occurrence of BNAN on day-3 embryos. Therefore, even if the more complex structure of the day-3 embryos is unfavorable for nuclear assessment, one could argue that a day-2 embryo with BNAN has a higher likelihood of developing to a day-3 embryo with BNAN. Indeed, in this study we also found that the implantation rate was much lower when the transferred blastocysts derived from embryos with BNAN on both days 2 and 3.

Assessment of blastomere nucleation in an embryo is a fast procedure. Although the presence of obscuring fragments and blastomere overlap might difficult the assessment of the blastomere nucleus, these obstacles can be solved by rolling the embryo on the bottom of the dish and by focus depth alteration, respectively (Saldeen & Sundström, 2005). To guarantee that the blastomere is in fact lacking a nucleus, embryos should be evaluated often at consistent time intervals (Moriwaki *et al*., 2004). However, embryo morphological assessment is limited to once a day, since frequent removal of embryos from the incubator may change culture medium temperature and pH (Desai *et al.*, 2014). Therefore, time-lapse may be considered as the optimum technique to assess blastomere nucleation. Nevertheless, the evaluation of nuclear status using simple light microscopy has proven to be predictive of embryo developmental capacity.

Nucleation asynchrony in early cleavage-stage embryos is a natural event. It is known that the development of blastomere nuclei involves different phases, during which the nuclei may be visible or not. It is also possible that nucleation asynchrony in a four-cell embryo results in further cleavage asynchrony (i.e., to a five-, six-, or seven-cell embryo instead of to an eight-cell embryo) (Saldeen & Sundström, 2005). Since nuclear formation is a dynamic process, it might be argued that the evaluation of nuclear status relying on short time interval observations is misleading. Even though it is not possible to state that the occurrence of BNAN is merely a cell-cycle artefact, or indeed a cell with no nucleus, this study demonstrates that embryos presenting such cells at the moment of embryo evaluation on days 2 and 3 using the Istanbul consensus (44h±1h and 68h±1h post ICSI) showed worse prognosis. There is also a possible relationship between blastomere nucleation and aneuploidy that cannot be excluded, but as no preimplantation diagnostic tests were made, we could not correlate these two phenomena.

The main limitation of this study relates to its design. The ideal design would include only cycles with single embryo transfer, or multiple embryo transfer cases presenting embryos with the same BNAN category, otherwise, it is impossible to know which embryo implanted. However, the selection of cycles with single embryo transfer, or multiple embryo transfer presenting all the embryos the same BNAN category would result in a reduced number of cases that would prevent statistical significance. Considering that both mononucleated and BNAN embryos were transferred in multiple embryo transfer cycles, we could suggest that the presence of BNAN is associated with lower likelihoods of successful implantation, even when mononucleated embryos are transferred along with embryos with BNAN. Additionally, despite the somewhat large number of embryos evaluated in this study, the relatively rare occurrence of BNAN in embryos that were actually transferred limits the experimental analysis of this feature. Thus, one could not rule out the possibility that the association between BNAN and implantation is related to an insufficient sample size.

In conclusion, careful nuclear observation, taking into account not only the presence of blastomere multinucleation but also the absence of nucleus, should be part of the strategies used for embryo selection for transfer and cryopreservation.
